# CD27^-^CD38^low^CD21^low^ B-Cells Are Increased in Axial Spondyloarthritis

**DOI:** 10.3389/fimmu.2021.686273

**Published:** 2021-06-08

**Authors:** Rick Wilbrink, Anneke Spoorenberg, Suzanne Arends, Kornelis S. M. van der Geest, Elisabeth Brouwer, Hendrika Bootsma, Frans G. M. Kroese, Gwenny M. Verstappen

**Affiliations:** Department of Rheumatology and Clinical Immunology, University of Groningen, University Medical Center Groningen, Groningen, Netherlands

**Keywords:** axial spondyloarthritis, ankylosing spondylitis, B-cells, autoimmunity, Sjögren’s syndrome, CD21low B-cells

## Abstract

B-cells have received little attention in axial spondyloarthritis (axSpA) and for this reason their role in pathogenesis remains unclear. However, there are indications that B-cells may be involved in the disease process. Our objective was to obtain insights into the composition of the peripheral B-cell compartment of axSpA patients compared to healthy donors (HD) and patients with primary Sjögren’s syndrome (pSS), a typical B-cell-associated autoimmune disease. Special emphasis was given to CD27-negative B-cells expressing low levels of CD21 (CD21^low^ B-cells), since this subset is implicated in autoimmune diseases with strong involvement of B-cells. Transitional B-cells (CD38^hi^) were excluded from the analysis of the CD27^-^CD21^low^ B-cell compartment. This study included 45 axSpA patients, 20 pSS patients and 30 HDs. Intriguingly, compared to HDs the frequency of CD27^-^CD38^low^CD21^low^ B-cells was significantly elevated in both axSpA and pSS patients (P<0.0001 for both comparisons). The frequency of CD27^-^CD38^low^CD21^low^ B-cells expressing the activation-induced immune markers T-bet and CD11c was decreased in axSpA patients compared to HDs. A higher proportion of CD27^-^CD38^low^CD21^low^ B-cells expressed the chemokine receptor CXCR3 in axSpA compared to HDs, suggestive for active involvement of these cells in an inflammatory process. The frequency of CD27^-^CD38^low^CD21^low^ B-cells in axSpA patients correlated positively with age and erythrocyte sedimentation rate. Furthermore, axSpA patients with extra-skeletal manifestations (ESM) showed increased frequencies of CD27^-^CD38^low^CD21^low^ B-cells compared to patients without ESM. In conclusion, our findings are suggestive of active B-cell involvement in the pathogenesis of axSpA, against prevailing dogma.

## Introduction

Axial spondyloarthritis (axSpA) is a chronic immune-mediated disease characterized by inflammation mainly present in sacroiliac joints (SI) and spine (1). To a lesser extent, also peripheral joints and entheses are involved. AxSpA can be classified into two sub-types, ankylosing spondylitis (AS) and non-radiographic axSpA (nr-axSpA). In contrast to AS, patients with nr-axSpA have not (yet) developed detectable damage of the SI joints, as shown by X-ray. A substantial percentage of axSpA patients will also develop extra-skeletal manifestations (ESM), such as uveitis, psoriasis and inflammatory bowel disease (2). The innate immune system and T-cells play a critical role in axSpA pathogenesis with a pivotal function for the IL-23/IL-17 axis (3). These cells and cytokines, together with the presence of human leukocyte antigen (HLA) B27, are considered as the main factors driving the pathophysiology of axSpA. Also, the gut-joint axis of inflammation might play an important role, since spondyloarthropathies are strongly associated with gut inflammation (4). Thus far, B-cells have received little attention regarding their (potential) role in the pathogenesis of axSpA. The lack of literature is largely rooted in absence of conventional disease-defining autoantibodies such as rheumatoid factor and anti-citrullinated protein antibodies (ACPAs) that are seen in rheumatoid arthritis (RA). Nonetheless, there are some indications that B-cells may be involved in the pathogenesis of axSpA. A clinical trial by Song et al. revealed positive clinical effects of B-cell depletion therapy with rituximab in a subcategory of axSpA patients (5). Also, axSpA patients may exhibit reduced frequencies of blood CD19^+^CD27^+^ memory B-cells (6). Furthermore, several antibodies to microbial and autoantigenic targets have been observed in axSpA [reviewed by Quaden et al. (7)], in particular autoantibodies against CD74 (MHC class II invariant chain) (8–10). In addition, an antinuclear antibody (ANA) titer of 1:80 or higher was present in 20% of AS patients (11). Finally, GWAS studies revealed that a polymorphism in *BACH2* is associated with AS. *BACH2* is a transcription factor that is, among others, implicated in negative selection resulting in less stringent depletion of newly generated B-cells (12, 13).

A B-cell subset that has been particularly associated with chronic inflammation and autoreactivity in recent years is characterized by low expression of the complement receptor CD21 (CD21^low^ B-cells) (14). These CD21^low^ B-cells are enriched in patients with several systemic autoimmune diseases such as RA, systemic lupus erythematosus (SLE) and primary Sjögren’s syndrome (pSS), as well as in patients with Common Variable Immunodeficiency Disorder (CVID) (15–17). CD21^low^ B-cells are a heterogeneous population of cells, comprised of both CD27-positive and CD27-negative B-cells. In healthy individuals (15), as well as in pSS patients (16), a substantial proportion of CD27^-^CD21^low^ B-cells are switched memory cells. However, in RA and CVID patients, these cells are predominantly naïve B-cells, expressing unmutated IgM (15). At least part of the CD21^low^ B-cells are considered to represent anergic, autoreactive B-cells, and in patients with pSS, RA and CVID these cells express auto-antibodies against nuclear and cytoplasmic antigens (15, 16). These anergic B-cells fail to become activated through conventional B-cell receptor (BCR) and CD40 signaling (15). At the same time, stimulation of CD21^low^ B-cells *via* toll-like receptors (TLR) does, however, induce a proliferative response in a proportion of these cells (15, 16, 18). Irrespective of differences in CD27 expression, a proportion of the CD21^low^ B-cells in healthy and diseased individuals appear to be in an activated state exhibiting homing capacity to sites of inflammation [reviewed by Thorarinsdottir et al. (14)].

In order to explore the potential role of B-cells in the pathogenesis of axSpA we analyzed the composition and phenotype of circulating B-cells in these patients. Special emphasis was given to CD21^low^ B-cells. We compared B-cells from axSpA patients not only to B-cells from healthy donors (HD), but also to B-cells from patients with pSS, a typical B-cell mediated autoimmune disease that is characterized by B-cell hyperactivity (19, 20). Finally, we investigated whether possible changes in the B-cell compartment were associated with clinical parameters in axSpA patients.

## Methods

### Patients and Healthy Donors

Peripheral blood mononuclear cells (PBMCs) were obtained from 45 axSpA patients, 20 age-matched pSS patients and 30 HDs, age- and sex-matched to the axSpA group. We included consecutive axSpA patients from the Groningen-Leeuwarden axial spondyloarthritis (GLAS) cohort (21). The GLAS cohort is an on-going prospective longitudinal observational cohort study, with a fixed protocol of follow-up visits. All patients fulfilled the ASAS criteria for axSpA. Patients with axSpA using biological disease-modifying anti-rheumatic drugs (DMARDs) within six months prior to inclusion were excluded. As disease control group, we included 20 consecutive pSS patients participating in the REgistry of Sjögren syndrome in UMCG LongiTudinal (RESULT) cohort. These patients fulfilled the 2016 ACR-EULAR classification criteria for pSS. Patients with pSS were not treated with DMARDs or immunosuppressants at the time of inclusion. HD samples were obtained *via* Sanquin Blood Supply Foundation, Netherlands, n=20, and the SENEX healthy aging cohort of the University Medical Center Groningen, Netherlands, n=10 (22). All participants of the study provided informed consent, in accordance with the Declaration of Helsinki. The study was approved by the medical research ethics committee of the Medical Center Leeuwarden (RTPO 364/604). Patient and HD characteristics are summarized in **Table 1**. Of the axSpA patients, 80% were classified as and 20% as nr-axSpA. There were no differences in patient characteristics between AS and nr-axSpA patients.

**Table 1:** Characteristics of axSpA, pSS patients and healthy donors.

	axSpA	pSS	HD
Number of patients	45	20	30
Age (years)	49 ± 13	51 ± 12	48 ± 21
Male gender (%)	62	20	63
Symptom duration (years)	23 (15)	11 (9)	
ESR (mm/hr)	13 (15.8)	20 (15.5)	
CRP (mg/L)	2.9 (5.2)	1.5 (3.3)	
IgG (g/L)		14.1 (5.1)	
History of biologic DMARD use (%)	42.2	30	
**AxSpA specific features:**			
AS (%)	80		
HLA-B27 positive (%)	93		
ASDAS	2.5 ± 1.0		
BASDAI	4.3 ± 2.4		
History of:			
Uveitis	16 (36)		
Inflammatory bowel disease	4 (9)		
Psoriatic arthritis	3 (7)		
>1 ESM	19 (37)		
History of:	13 (29)		
Peripheral arthritis	6 (13)		
Enthesitis	5 (11)		
Dactylitis	3 (7)		
≥1 PM	13 (29)		
**pSS specific features:**			
ESSPRI		6.3 ± 2.2	
ESSDAI		3 (6)	
Anti-Ro/SSA positive (%)		70	
Anti-La/SSB positive (%)		50	
Rheumatoid factor positive (%)		60	
Schirmer’s test (mm/5min)		3.0 (12.0)	
Ocular staining score		2.0 (5.0)	
UWSF (mL/min)		0.1 (0.3)	
Focus score		2.8 ± 2.0	

Data are presented as number of patients (%), mean ± SD or median (IQR); AS, Ankylosing spondylitis; HLA-B27, Human leukocyte antigen B27; DMARD, Disease modifying antirheumatic drugs; BASDAI, Bath Ankylosing Spondylitis Disease Activity Index; ASDAS, Ankylosing Spondylitis Disease Activity Score; ESM, extra skeletal-manifestations; PM, peripheral manifestations; ESSPRI, EULAR Sjögren’s Syndrome Patient Reported Index; ESSDAI, EULAR Sjögren’s syndrome disease activity index; Anti-SSA, Anti-Sjögren’s-syndrome-related antigen A autoantibodies; Anti-SSB, Anti-Sjögren’s-syndrome-related antigen B autoantibodies; UWSF, unstimulated whole saliva flow; Focus score in parotid gland tissue.

### Flow-Cytometry Analysis

PBMCs from patients and HDs were phenotypically analyzed by 14-color flow-cytometry. Cryopreserved cells were thawed and resuspended in phosphate buffer saline (PBS), supplemented with 0,5% bovine serum albumin and 2mM ethylenediaminetetraacetic acid. Two million cells were stained for surface markers described in **Supplementary Table 1**. After washing with PBS, cells were stained with a fixable viability dye (eFluor 506; eBioscience). Finally, PBMCs were washed, fixed and permeabilized using the Foxp3 fixation/permeabilization buffer set (eBioscience) to allow intracellular staining of T-bet. Data were acquired on a BD LSR-II flow-cytometer and analyzed using FlowJo software 10.7 (Tree Star, Ashland, Oregon). Gates were based on fluorescence-minus-one (FMO) controls.

### Statistics

All data were statistically analyzed using Statistical Package for the Social Sciences (SPSS) and Graphpad Prism (v9.0.1). Differences between groups were tested using the Independent Samples t-test or Mann-Whitney U test, depending on the distribution of variables. Associations between CD21^low^ B-cells and clinical parameters were explored using the Pearson or Spearman correlation coefficient, depending on the distribution of variables. P-values<0.05 were considered statistically significant.

## Results

### Normal Frequencies of Naïve and Memory B-Cells, but Expansion of Plasmablasts in axSpA

**Figure 1:**
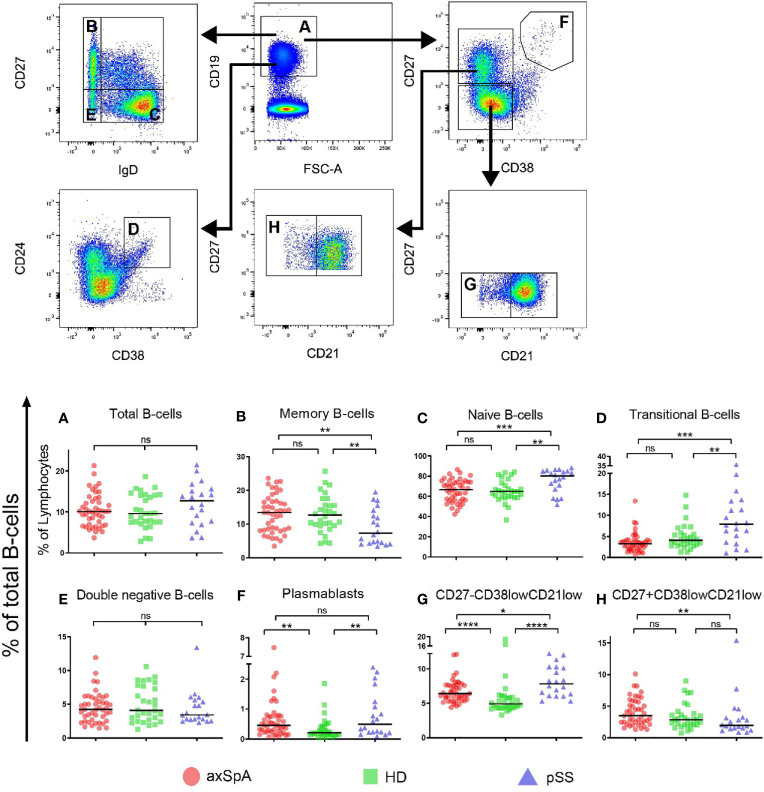
Frequencies of total B-cells and B-cell subsets in patients with axSpA, pSS and healthy donors. Global gating strategy is shown with arrows pointing towards the next level of gating. Frequencies of **(A)** total B-cells (CD19^+^), relative to total lymphocytes, and the following B-cell subsets, relative to total B-cells, are shown: **(B)** memory B-cells (CD27^+^IgD^-^), **(C)** double negative (CD27^-^IgD^-^) B-cells, **(D)** naïve (CD27^-^IgD^+^) B-cells, **(E)** plasmablasts (CD27^+^CD38^hi^), **(F)** transitional (CD24^hi^CD38^high^) B-cells, **(G)** CD27^+^CD38^low^CD21^low^ B-cells and **(H)** CD27^-^CD38^low^CD21^low^ B-cells. Patients with axial spondyloarthritis (axSpA; n = 45) are indicated with red circles, healthy donors (HD; n = 30) are indicated with green squares and patients with primary Sjögren’s Syndrome (pSS; n = 20) are indicated with blue triangles. *P < 0.05, **P < 0.01, ***P < 0.001, ****P < 0.0001, ns, not significant and horizontal lines indicate the medians.

First, we studied the frequencies of total B-cells and classical naïve and memory subsets within the total CD19^+^ B-cell compartment in axSpA patients, pSS patients and HDs (gating strategy is shown in **Figure 1**). Patients with axSpA had a similar frequency of total B-cells (relative to all lymphocytes) as pSS patients and HDs (**Figure 1A**). The frequency of classical memory B-cells (CD27^+^IgD^-^) and naïve (CD27^-^IgD^+^) B-cells was comparable between axSpA patients and HDs (**Figures 1B, C**). In contrast, but as expected (23), in pSS patients the frequency of these classical memory cells was decreased and accompanied by an increased frequency of naïve B-cells. The frequency of transitional B-cells (CD24^hi^CD38^hi^) in patients with axSpA did not differ from HDs, but in pSS patients frequencies were higher compared to axSpA patients and HDs (**Figure 1D**). Patients with pSS further exhibited lower frequencies of marginal zone-like B-cells (CD27^+^IgD^+^) compared to axSpA patients and HDs (data not shown). The frequency of double negative B-cells (CD27^-^IgD^-^) was comparable among the three groups (**Figure 1E**). Interestingly, the frequency of plasmablasts (CD27^hi^CD38^hi^) was significantly higher in both patients with axSpA and patients with pSS compared to HDs (P<0.01 for both comparisons, **Figure 1F**).

### Enrichment of Circulating CD27^-^CD38^low^CD21^low^ B-Cells in axSpA Patients

Given its role in autoimmune diseases, special emphasis was given to CD21^low^ B-cells. To this end transitional B-cells (CD27^-^CD24^hi^CD38^hi^), which also express low levels of CD21, were excluded from the analysis. The remaining CD21^low^ B-cells comprised CD27^-^CD38^low^ and CD27^+^CD38^low^ B-cell fractions (**Figure 1**). In all patient groups and HDs, the majority of CD21^low^ B-cells belonged to the CD27^-^CD38^low^ compartment. We found that the frequencies of CD27^-^CD38^low^CD21^low^ B-cells (of total CD19^+^ B-cells) were significantly higher in both axSpA (median=6.4%, P<0.0001) and pSS patients (median=7.8%, P<0.0001), compared to HDs (median=4.9%) (**Figure 1G**). Frequencies of CD27^+^CD38^low^CD21^low^ B-cells (of total CD19^+^ cells) were, however, similar between axSpA patients and HDs but significantly reduced in pSS patients (**Figures 1H**). Thus, not only frequencies of plasmablasts but also CD27^-^CD38^low^CD21^low^ B-cells were significantly higher in patients with axSpA (and pSS) compared to HDs.

### CD27^-^CD38^low^CD21^low^ B-Cells of Patients With axSpA Display Phenotypical Heterogeneity

**Figure 2:**
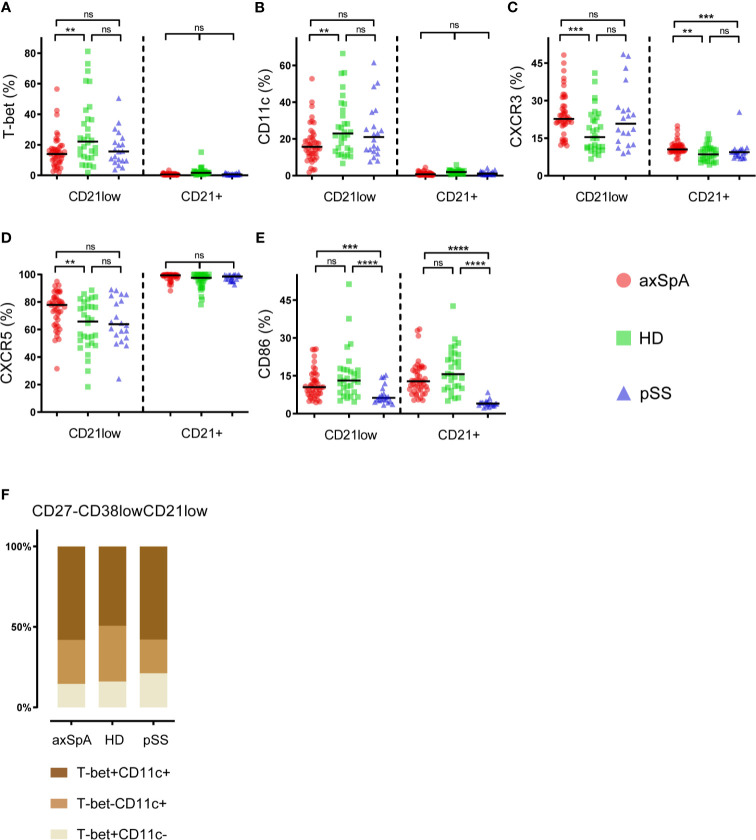
Phenotypical characteristics of CD27^-^CD38^low^CD21^low^ B-cells in patients with axSpA, pSS and healthy donors. The proportions of **(A)** T-Bet-positive, **(B)** CD11c-positive, **(C)** CXCR3-positive, **(D)** CXCR5-positive, **(E)** CD86-positive cells were analyzed within the CD27^-^CD38^low^CD21^+^ and CD27^-^CD38^low^CD21^low^ B-cell subsets. In addition, **(F)** an illustration of T-bet/CD11c co-expression by CD27^-^CD38^low^CD21^low^ B-cells is displayed. Patients with axial spondyloarthritis (axSpA; n = 45) are indicated with red circles, healthy donors (HD; n = 30) are indicated with green squares and patients with primary Sjögren’s Syndrome (pSS; n = 20) are indicated with blue triangles. **P < 0.01, ***P < 0.001, ****P < 0.0001, ns, not significant and horizontal lines indicate the medians.

A more detailed analysis was performed to study phenotypical characteristics of CD27^-^CD38^low^CD21^low^ B-cells (global gating strategy displayed in (**Supplementary Figure 1**). Comparison of CD27^-^CD38^low^CD21^low^ B-cells with their CD21^+^ counterpart (CD27^-^CD38^low^CD21^+^), showed that CD27^-^CD38^low^CD21^low^ B-cells express more frequently the immune markers T-Bet, CD11c and the chemokine receptor CXCR3, but less frequently CXCR5 (**Figure 2**). This was seen in both patient groups as well as in HDs. Expression of CD86 by CD27^-^CD38^low^CD21^low^ and CD27^-^CD38^low^CD21^+^ B-cells was similar in axSpA patients and HDs, however, pSS patients showed a significantly elevated frequency of CD86-positive CD27^-^CD38^low^CD21^low^ B-cells (P<0.0001).

When comparing the expression pattern of immune cell markers by CD27^-^CD38^low^CD21^low^ B-cells, we found differences among patients with axSpA, pSS and HDs (**Figures 2A–E**). The frequency of T-bet- and/or CD11c-expressing cells, often co-expressed (24), was significantly lower in axSpA patients compared to HDs. T-bet and/or CD11c-positive CD27^-^CD38^low^CD21^low^ B-cells also tended to be lower in pSS patients, although not significant. In both patient groups and HDs, roughly half of the T-bet- and/or CD11c-expressing cells co-expressed these markers (**Figure 2F**). Compared to HDs, both CXCR3- and CXCR5-positive CD27^-^CD38^low^CD21^low^ B-cells were significantly higher in patients with axSpA. Interestingly, also within their CD21^+^ counterpart (CD27^-^CD38^low^CD21^+^) the proportion of CXCR3^+^ cells was increased in axSpA patients compared to HDs and pSS patients. The frequency of CD86-positive cells within the CD27^-^CD38^low^CD21^low^ B-cell compartment was similar between axSpA patients and HDs, but was reduced in pSS patients. When examining the relation between T-bet expression by CD27^-^CD38^low^CD21^low^ B-cells and other immune markers we found that the proportion of T-bet^+^ B-cells was strongly associated with proportions of CD11c^+^, CXCR3^+^, and CD86^+^ B-cells within this subset in axSpA patients (**Supplementary Figure 2**).

**Figure 3:**
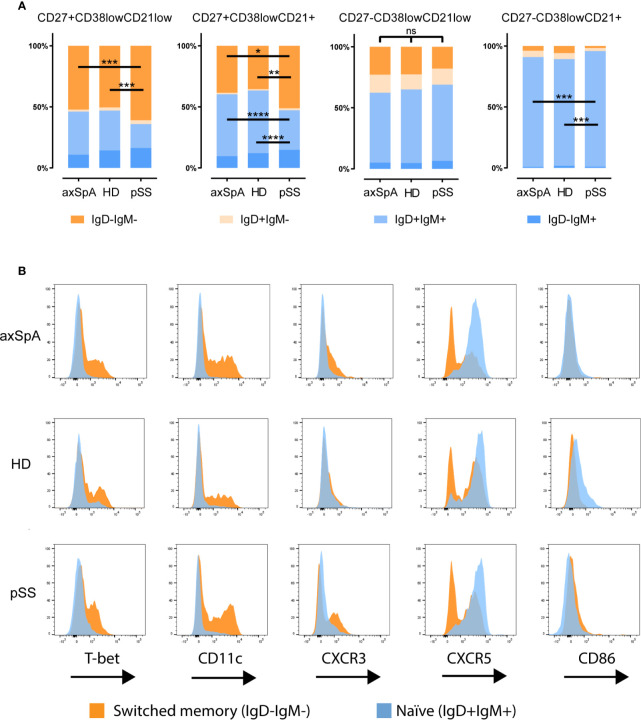
Distribution of immunoglobulins IgD and IgM by CD27CD38^low^CD21 B-cell compartment in patients with axSpA, pSS and HDs. Analysis of the distribution of immunoglobulins IgD and IgM **(A)** is shown for CD27^+^CD38^low^CD21^low^, CD27^+^CD38^low^CD21^+^, CD27^-^CD38^low^CD21^low^, CD27^-^CD38^low^CD21^+^ B-cells respectively. A more detailed analysis of the immune marker expression **(B)** by the naïve (IgD^+^IgM^+^) in orange and switched (IgD^-^IgM^-^) B-cell populations in blue shown as histogram plots comparing CD27^-^CD38^low^CD21^low^ B-cell populations of one axial spondyloarthritis patient, one primary Sjögren’s syndrome patient and a healthy donor. *P < 0.05, **P < 0.01, ***P < 0.001, ****P < 0.0001, ns, not significant and horizontal lines indicate the medians.

Since CD27 is not a definitive marker for discrimination between naïve and memory B-cells, we also investigated IgD and IgM expression patterns by CD27^-^CD38^low^CD21^low^ B-cells (**Figure 3A**). There was no difference in IgD/IgM distribution in this subset among study groups. Within the CD27^-^CD38^low^CD21^low^ fraction, most B-cells were naïve IgD^+^IgM^+^ (~60%) followed by IgM^-^IgD^-^ (switched) B-cells (~20%). Of note, the CD27^-^CD38^low^CD21^+^ B-cell counterpart contained hardly any switched cells. A more in depth analysis of variation in expression of the immune markers between switched and naïve cells within the CD27^-^CD38^low^CD21^low^ B-cell compartment of an axSpA patient is shown in **Figure 3B**, illustrating that expression of T-bet, CD11c, CXCR3, and absence of CXCR5 can all be attributed to the switched memory (IgD^-^IgM^-^) B-cell fraction.

Taken together, these results indicate that in axSpA patients CD27^-^CD38^low^CD21^low^ B-cells display a phenotype that is distinct from their CD21^+^ counterpart, similar to what we observed pSS patients and HDs. Subtle differences in the phenotype of CD27^-^CD38^low^CD21^low^ B-cells were present among the groups, with a significant increase in the frequency of CXCR3^+^ and CXCR5^+^ cells and a decrease in T-bet^+^ and CD11c^+^ cells in axSpA patients compared with HDs.

### Relation Between CD27^-^CD38^low^CD21^low^ B-Cells, Clinical Parameters and Disease Activity in axSpA

**Figure 4:**
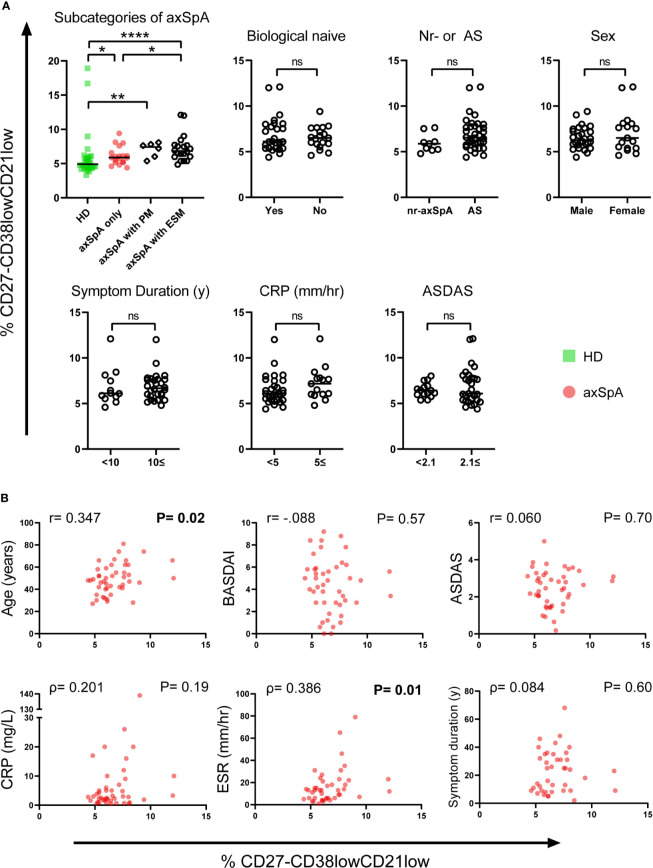
Relationship between frequencies of CD27^-^CD38^low^CD21^low^ B-cells and clinical parameters in patients with axSpA. **(A)** Patients with axial spondyloarthritis (axSpA) were divided into separate groups based on subcategories of axSpA and the following clinical parameters: Biological DMARD naïve, nr-axSpA or axSpA, sex, symptom duration, C-reactive protein (CRP) and Ankylosing Spondylitis Disease Activity Score (ASDAS). **(B)** To explore associations between the CD27^-^CD38^low^CD21^low^ B-cell compartment and several continuous clinical parameters in patients with axSpA, the frequency of CD27^-^CD38^low^CD21^low^ B-cells was plotted against the following clinical parameters: Age, Bath Ankylosing Spondylitis Disease Activity Index (BASDAI), ASDAS, CRP, erythrocyte sedimentation rate (ESR), symptom duration. Patients with axSpA(n=45) are indicated with red circles, healthy donors (HD; n = 30) are indicated with green squares. *P < 0.05, **P < 0.01, ****P < 0.0001, significant values in bold, ns, not significant and horizontal lines indicate the medians.

To study the clinical relevance of CD27^-^CD38^low^CD21^low^ B-cells in axSpA, we looked at various clinical parameters and their possible association with this particular B-cell subset (**Figure 4**).

When comparing axSpA patients with and without a history of ESM, including uveitis, psoriasis and inflammatory bowel disease (IBD), we observed a significantly higher frequency of CD27^-^CD38^low^CD21^low^ B-cells in patients with a known history of ESM (median=7.05%, P=0.02) compared to patients without ever having an ESM (median=5.9%). Still, axSpA patients without ESM displayed an increased frequency of CD27^-^CD38^low^CD21^low^ B-cells compared to HDs (P=0.01), as shown in **Figure 4A**. It is especially noteworthy that between axSpA patients with a history ESM and pSS patients the frequency of these B-cells did not vary (P=0.18, data not shown). We also compared axSpA patients without and with a history of peripheral manifestations including enthesitis, arthritis and dactylitis (without extra-skeletal manifestations) and observed no notable enrichment of CD27^-^CD38^low^CD21^low^ B-cells between groups (P=0.14).

The following categorical clinical parameters were not associated with enrichment of CD27^-^CD38^low^CD21^low^ B-cells in patients with axSpA: being naïve for biological DMARDs, non-radiographic disease, sex, symptom duration ≥10 years, C-reactive protein (CRP) ≥5 mg/L and high disease activity defined by the Ankylosing Spondylitis Disease Activity Score (ASDAS) ≥2.1 (**Figure 4A**). We found that age and erythrocyte sedimentation rate (ESR), as continuous variables, were both significantly correlated with the frequency of CD27^-^CD38^low^CD21^low^ B-cells in axSpA patients (P=0.02, P=0.01 respectively, **Figure 4B**). Interestingly, the correlation was driven by axSpA patients with a history of ESM (P=0.04) (data not shown). Of note, in HDs and pSS patients this subset was not correlated with age (P=0.36, P=0.28 respectively) and in pSS also not with ESR (P=0.75, data not shown). Continuous measures of diseases activity, such as Bath Ankylosing Spondylitis Disease Activity Index (BASDAI), ASDAS and CRP as well as symptom duration were not correlated with increased frequencies of CD27^-^CD38^low^CD21^low^ B-cells.

In summary, the frequency of CD27^-^CD38l^ow^CD21^low^ B-cells in axSpA patients was associated with age and ESR. Furthermore, axSpA patients with a history of ESM have a greater frequency of circulating CD27^-^CD38l^ow^CD21^low^ B-cells than patients presenting with only axial involvement (with or without peripheral manifestations).

## Discussion

In this cross-sectional study, we observed a higher frequency of circulating CD27^-^CD38^low^CD21^low^ B-cells in patients with axSpA, compared to healthy donors. A comparable rise in CD27^-^CD38^low^CD21^low^ B-cells was noted in patients with pSS, a typical B-cell-associated autoimmune disease. The phenotype of these cells was broadly similar among axSpA patients, pSS patients and HDs, although subtle differences existed. Since this particular CD21^low^ B-cell subset is associated with autoreactivity (15, 16, 25), an elevated frequency of CD27^-^CD38^low^CD21^low^ B-cells, together with the observed enrichment of circulating plasmablasts, and the association of these CD27^-^CD38^low^CD21^low^ B-cells with clinical parameters may reflect that also in axSpA patients B-cells are involved in the pathogenesis, against prevailing dogma.

The CD27^-^CD38^low^CD21^low^ B-cell compartment is comprised of both naïve and memory B cells (26). Naïve CD21^low^ B-cells are thought to represent anergic B cells (15), potentially resulting from defective central B-cell tolerance and failure of receptor editing (27, 28), whereas memory CD21^low^ B-cells might either portray recent antigen experienced B-cells (29–31) or exhausted memory B-cells (18). Here we show that also in axSpA patients the CD21^low^ B-cell compartment is heterogeneous in terms of naïve and memory cells. In our study the IgD and IgM expression profiles revealed that most CD27^-^CD38^low^CD21^low^ B-cells in axSpA patients (as well as in pSS patients and HDs) could be defined as unswitched B-cells (IgD^+^IgM^+^), representing naïve CD21^low^ B-cells. However, we also observed a substantial proportion of switched memory cells (IgD^-^IgM^-^) in the CD27^-^CD38^low^CD21^low^ B-cell compartment, in contrast to the CD27^-^CD38^low^CD21^+^ B-cell subset.

Further phenotypical analysis showed that a fraction of the CD27^-^CD38^low^CD21^low^ B-cell subset in both groups of patients and HDs expresses T-bet and/or CD11c, and most of them (>50%) co-express these markers. T-bet/CD11c expressing B-cells were confined to the CD21^low^ subset and are not found among CD21^+^ B-cells, in line with other studies (32–34). T-bet/CD11c is expressed by activated, antigen-experienced memory cells (32–34) and higher frequencies of T-bet^+^CD11c^+^ double expressing B-cells are found in humans following infection or vaccination (29, 35–37). T-bet^+^CD11c^+^ B-cells are enriched in settings of chronic stimulation, including autoimmunity, and produce polyclonal antibodies against self-antigens (14, 24). In line with the memory phenotype of T-bet/CD11c expressing B-cells, we observed that within the CD27^-^CD38^low^CD21^low^ B-cell subset most of the T-bet^+^ and/or CD11c^+^ cells were indeed class-switched cells, in all groups of individuals. Furthermore, the associated expression of the co-stimulatory molecule CD86 with T-bet on CD27^-^CD38^low^CD21^low^ B-cells in axSpA patients indicates that these B-cells are activated cells, which are prone to differentiate into antibody secreting cells (ASC) (32, 34, 38). In line with this notion, we observed a higher frequency of plasmablasts in the peripheral blood of axSpA patients, possibly related to increased levels of immunoglobulins, in particular IgA, in the serum of these patients (39–41). Interestingly, a single nucleotide polymorphism in the gene encoding for T-bet (*TBX21*) is associated with AS (42), illustrating a role for T-bet in the disease, not only in T-cells and NK-cells, but possibly also in B-cells. To summarize, the CD21^low^ B-cell compartment in axSpA is composed of naïve and memory B-cells, of which a minor, potentially pathogenic, subpopulation expresses T-bet and/or CD11c that may represent ASC precursors. Whether these cells are directed against self- or non-self-antigens in axSpA patients remains to be shown.

When comparing of the frequency of T-bet^+^ and CD11c^+^ cells within the CD27^-^CD38^low^CD21^low^ B-cells compartment between study groups, we found that patients with axSpA displayed a slightly, but significantly reduced proportion of T-bet^+^ or CD11c^+^ B-cells compared with HDs. The lower frequency of T-Bet^+^ and/or CD11c^+^ B-cells in the peripheral blood of axSpA patients may be explained by enhanced differentiation into plasmablasts and/or migration to sites of inflammation. The latter is supported by the finding that a high proportion of CD27^-^CD38^low^CD21^low^ B-cells in axSpA did express the chemokine receptor CXCR3, responsible for homing of cells to inflamed tissue. In addition, our finding that in axSpA patients the level of CD27^-^CD38^low^CD21^low^ B-cells correlated with ESR, an indicator for more long-term inflammation, is suggestive for involvement of B-cells in the inflammatory process (43). Intriguingly, plasmablast infiltration and ectopic lymphoid tissue formation with follicular dendritic cell networks have been found in the synovial membranes of inflamed hips of AS patients (44, 45). CD20^+^ B-cells are also present in the zygapophyseal joints in patients with active disease (46). Furthermore, a study in a mouse model of AS by Kaai et al., reported infiltrates of inflammatory cells at the edge of intervertebral discs and lymphoid aggregates in the bone marrow (47). CD21^low^ B-cells are also found in the synovial fluid of RA patients (48). At these sites they are associated with joint destruction possibly mediated by their capacity to secrete osteoclastogenic factor receptor activator of the nuclear factor kB ligand (RANKL). Hence, the capacity of B-cells and in particular CD21^low^ B-cells to potentially migrate towards either skeletal or extra-skeletal sites of inflammation might be of particular relevance in the pathogenesis of axSpA (2). An earlier study reported increased levels of circulating CD21^low^ B-cells in Crohn’s disease, which is an ESM and sub-category of IBD (49). In addition, infiltrations of B-cells in intestinal tissue are found in IBD patients (50). Also in other ESM, namely psoriasis and uveitis, subtle changes of the B-cell compartment and tissue infiltrations have been noted (51, 52). Our finding that axSpA patients with a history of ESM show enriched frequencies of CD27^-^CD38^low^CD21^low^ B-cells support the notion that disturbances found in the B-cell compartment are suggestive of an active role of B-cells in (some of) these ESM in axSpA.

In conclusion, we showed that the frequencies of CD27^-^CD38^low^CD21^low^ B-cells are elevated in axSpA, similar as observed in an established B-cell-associated autoimmune disease, pSS. Since CD27^-^CD38^low^CD21^low^ B-cells are implicated in various autoimmune and inflammatory diseases, we postulate that there is a hitherto underestimated role for B-cells in the pathogenesis of axSpA. B-cells are known to exhibit a wide repertoire of effector functions, including production of antibodies, antigen presentation and secretion of cytokines (7, 53). The role of B-cells in axSpA pathogenesis is not clear yet, and might well be beyond the production of (auto)antibodies. Of particular relevance in axSpA is the ability of B-cells to produce TNF-α, IL-23 crucial cytokines in the pathogenesis of axSpA (54–57). Future studies are needed to elucidate the functional capabilities of these CD27^-^CD38^low^CD21^low^ B-cells, including their (auto)antibody secreting capacity, in axSpA patients. Furthermore, it is important to determine whether CD27^-^CD38^low^CD21^low^ B-cells are also present at inflammatory sites of the disease.

## Data Availability Statement

The raw data supporting the conclusions of this article will be made available by the authors, without undue reservation.

## Ethics Statement

The studies involving human participants were reviewed and approved by the medical research ethics committee of the Medical Center Leeuwarden (RTPO 364/604). The patients/participants provided their written informed consent to participate in this study.

## Author Contributions

The concept of the study was developed by GV, FK, AS, and RW. Patient and healthy donor recruitment was done by AS, EB, KG, and HB. Data were obtained by RW and GV. Data were analyzed by RW, GV and SA. RW, GV, AS, and FK were involved in data analysis and interpretation of the results. RW wrote the first draft of the manuscript. All authors contributed to the article and approved the submitted version.

## Conflict of Interest

AS has received grant/research support from Abbvie, Pfizer, Union Chimique Belge (UCB), Novartis and acted as a consultant for Abbvie, Pfizer, MSD, UCB, Lilly and Novartis. SA has received grant/research support from Pfizer. KG has received a speaker fee from Roche. EB has received consultancy and speaker fees from Roche. HB has received unrestricted grants from Bristol Myers Squibb (BMS) and Roche, consultant for BMS, Roche, Novartis, MedImmune, UCB, speaker for BMS and Novartis. FK has received unrestricted grants from BMS, is consultant for BMS, speaker for BMS Roche and Jannsen-Cilag.

The remaining authors declare that the research was conducted in the absence of any commercial or financial relationships that could be construed as a potential conflict of interest.
