# Supplementary material for: CD27-CD38lowCD21low B-Cells Are Increased in Axial Spondyloarthritis
Source: Front Immunol. 2021 Jun 8;12:686273. doi: 10.3389/fimmu.2021.686273 (PMC8217653; doi:10.3389/fimmu.2021.686273)
Supplement: Supplementary file 1 [file DataSheet_1.zip › Supplementary Figure legends and supplementary Table 1.docx]

**SUPPLEMENTARY MATERIALS**

**Supplemental Figure 1. Global gating strategy to investigate the peripheral B-cell compartment**. Gating strategy shown is an example of PBMCs from a patient with axial spondyloarthritis. First, live cells were gated and consecutively single cells, lymphocytes, total (CD19^+^) B-cells and B-cell subpopulations. Lines with arrowheads are drawn between consecutive gates.

**Supplemental Figure 2. The association between T-bet-positive CD27^-^CD38^low^CD21^low^ B-cells and CD27^-^CD38^low^CD21^low^ B-cells positive for other immune markers in axSpA patients.** Associations between T-bet-positive CD27^-^CD38^low^CD21^low^ B-cells and CD27^-^CD38^low^CD21^low^ B-cells positive for other immune markers are shown. These markers include CD11c, CXCR3, CXCR5 and CD86. Scatter plots include percentages of cells within the CD27^-^CD38^low^CD21^low^ B-cell compartment of patients with axial spondyloarthritis (axSpA). Patients with axSpA (n=45) are indicated with red circles. Correlations between CD21^low^ B-cells and clinical parameters were explored using the Pearson or Spearman correlation coefficient, depending on the distribution of variables.

| Supplementary Table 1. Antibodies for anti-human immune markers staining | | |
| --- | --- | --- |
| Immune marker | Fluorochrome | Company |
| CD19 | BV786 | BD Bioscience |
| CD20 | Af700 | BD Bioscience |
| CD27 | APC | eBioscience |
| CD21 | BV421 | BD Bioscience |
| IgD | BUV395 | BD Bioscience |
| IgM | PerCP-Cy5.5 | eBioscience |
| T-bet* | PE | Biolegend |
| CD11c | BV605 | BD Bioscience |
| CXCR3 | PE-Cy7 | BD Bioscience |
| CXCR5 | BB515 | BD Bioscience |
| CD38 | APC-eF780 | eBioscience |
| CD86 | BV711 | BD Bioscience |
| CD24 | PE-D594 | Biolegend |
| CD10 | BUV737 | BD Bioscience |
| *Staining for T-bet was performed after fixation and permeabilization of the cells | | |
