# Supplementary figures and images for: CD27-CD38lowCD21low B-Cells Are Increased in Axial Spondyloarthritis
Source: Front Immunol. 2021 Jun 8;12:686273. doi: 10.3389/fimmu.2021.686273 (PMC8217653; doi:10.3389/fimmu.2021.686273)

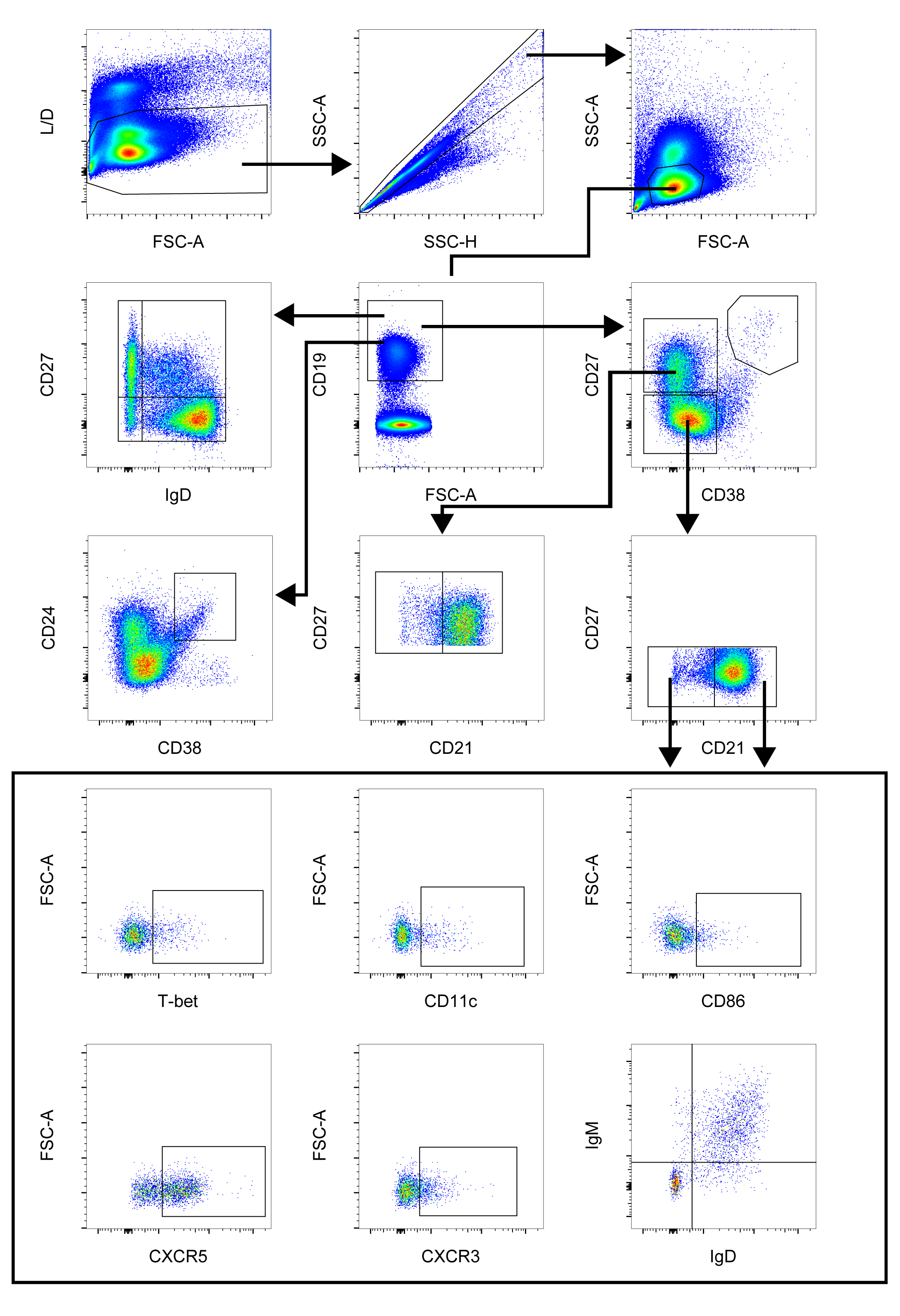

Supplement: Supplementary file 1 [file DataSheet_1.zip › Supplementary Figure 1.tif]

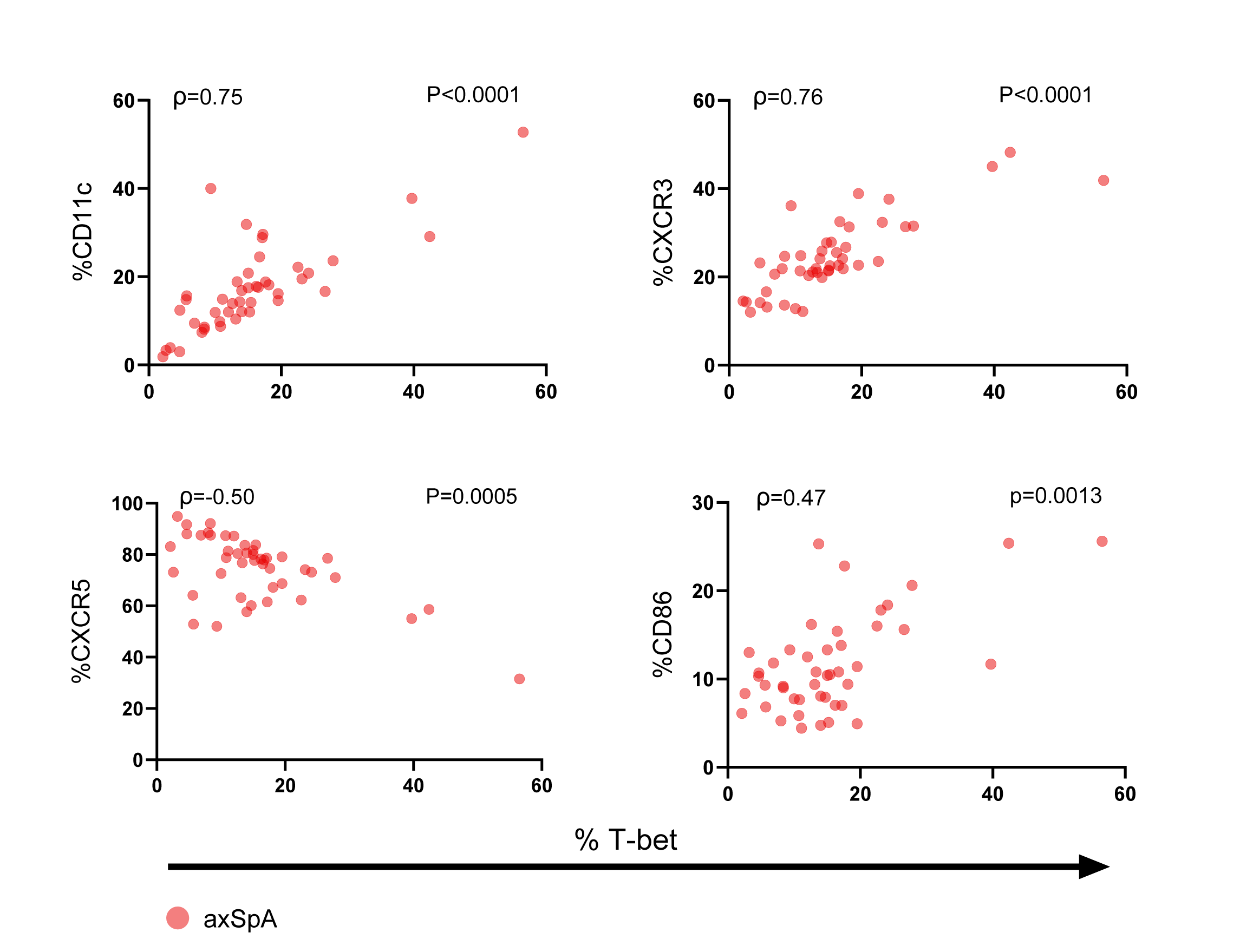

Supplement: Supplementary file 1 [file DataSheet_1.zip › Supplementary Figure 2.tif]
